# Profiling grapevine trunk pathogens *in planta*: a case for community-targeted DNA metabarcoding

**DOI:** 10.1186/s12866-018-1343-0

**Published:** 2018-12-14

**Authors:** Abraham Morales-Cruz, Rosa Figueroa-Balderas, Jadran F. García, Eric Tran, Philippe E. Rolshausen, Kendra Baumgartner, Dario Cantu

**Affiliations:** 1 Department of Viticulture and Enology, University of California Davis, One Shields Ave, Davis, CA 95616 USA; 2 Department of Botany and Plant Sciences, University of California, Riverside, CA 92521 USA; 3 United States Department of Agriculture, Agricultural Research Service, Crops Pathology and Genetics Research Unit, Davis, CA 95616 USA

**Keywords:** High-throughput DNA sequencing, Amplicon sequencing, Metagenomics, Grapevine trunk diseases, Esca, Eutypa dieback, Botryosphaeria dieback, Phomopsis dieback

## Abstract

**Background:**

DNA metabarcoding, commonly used in exploratory microbial ecology studies, is a promising method for the simultaneous *in planta*-detection of multiple pathogens associated with disease complexes, such as the grapevine trunk diseases. Profiling of pathogen communities associated with grapevine trunk diseases is particularly challenging, due to the presence within an individual wood lesion of multiple co-infecting trunk pathogens and other wood-colonizing fungi, which span a broad range of taxa in the fungal kingdom. As such, we designed metabarcoding primers, using as template the ribosomal internal transcribed spacer of grapevine trunk-associated ascomycete fungi (GTAA) and compared them to two universal primer widely used in microbial ecology.

**Results:**

We first performed in silico simulations and then tested the primers by high-throughput amplicon sequencing of (i) multiple combinations of mock communities, (ii) time-course experiments with controlled inoculations, and (iii) diseased field samples from vineyards under natural levels of infection. All analyses showed that GTAA had greater affinity and sensitivity, compared to those of the universal primers. Importantly, with GTAA, profiling of mock communities and comparisons with shotgun-sequencing metagenomics of field samples gave an accurate representation of genera of important trunk pathogens, namely *Phaeomoniella*, *Phaeoacremonium*, and *Eutypa*, the abundances of which were over- or under-estimated with universal primers.

**Conclusions:**

Overall, our findings not only demonstrate that DNA metabarcoding gives qualitatively and quantitatively accurate results when applied to grapevine trunk diseases, but also that primer customization and testing are crucial to ensure the validity of DNA metabarcoding results.

**Electronic supplementary material:**

The online version of this article (10.1186/s12866-018-1343-0) contains supplementary material, which is available to authorized users.

## Background

Grapevine trunk diseases affect the longevity and productivity of grapevines (*Vitis vinifera*) in all major growing regions of the world [1–4]. They are caused by numerous species of fungi that infect and damage the wood, causing chronic infections [5–7]. Among the most common grapevine trunk diseases are Eutypa dieback (primarily caused by *Eutypa lata*), Esca (primarily caused by *Phaeoacremonium minimum*, *Phaeomoniella chlamydospora*, and *Fomitiporia* spp.), Botryosphaeria dieback (primarily caused by *Neofusicoccum parvum*, *Diplodia seriata*, among other fungi in the Botryosphaeriaceae family), Phomopsis dieback (primarily caused by *Diaporthe ampelina*), and Black foot (caused by *Cylindrocarpon*, *Campylocarpon*, and *Ilyonectria* spp.) [4, 8–11]. Because of the characteristic mixed infections, trunk diseases represent a disease complex [12, 13]. In addition to infections of pruning wounds by airborne and splash-dispersed spores, trunk pathogens may be introduced to a healthy vineyard by asymptomatic propagation material. Fungi associated with grapevine trunk diseases have been found in rootstock mother-plants, rooted rootstock cuttings, bench-grafts, and young grafted vines [14–16]. The presence of multiple species in the same vine complicates disease diagnosis and, consequently, proper timing of practices to limit infection in the vineyard and to propagate clean nursery stock.

Taxonomic identification of fungi associated with grapevine wood is currently done by the following steps: (i) plating grapevine woody tissue on nutrient-rich agar plates, (ii) hyphal-tip colony isolation to pure cultures, (iii) DNA extraction from fungal mycelium, (iv) PCR amplification of taxonomically informative loci, such as the nuclear ribosomal internal transcribed spacer (ITS), elongation factor, and β-tubulin, and (v) comparisons of amplicon sequences with sequence databases [17–19]. PCR-based diagnostics represent a significant improvement compared to traditional approaches that depend on morphological features for species identification and, thus, require skilled expertise in mycology [20]. However, these approaches still require an initial culturing step, which may limit the detection of slow-growing fungi. Alternatively, with species or genus-specific markers, PCR could be used to determine *in planta* the presence of certain species, thereby skipping the culturing step [21, 22]. One limitation of this approach, however, is that it may not detect all trunk pathogens in a given sample [23, 24]. Indeed, certain combinations of fungi may be important in the severity of symptom expression [25].

Because trunk pathogens cause mixed infections, attempts have been made to characterize the composition of the trunk-pathogen community. For example, finger-printing techniques like Automated Ribosomal Intergenic Spacer Analysis (ARISA) [26] and Single-Strand Conformation Polymorphism (SSCP) [27, 28] have been used to compare fungal communities among different samples of grapevine wood, although these do not identify trunk pathogens to the species level.

Quantitative PCR (qPCR) has also been applied to the detection of grapevine trunk pathogens, including strategies that allow the monitoring of multiple species simultaneously [21, 22, 24, 29, 30]. A DNA macroarray system, based on reverse dot-blot hybridization containing oligonucleotides complementary to portions of the β-tubulin locus, was developed for species-level identification, specifically for detection of trunk pathogens that cause Young vine decline [23]. We previously described a strategy, based on untargeted shotgun sequencing of metagenomic DNA and RNA, to detect and quantify trunk pathogens *in planta* simultaneously [13]. Despite clear advantages over other approaches, this method still has its limitations, such as relying on assembled genomes, as well as costly library preparation and computationally intensive analyses.

DNA metabarcoding, which has been used extensively for the analysis of microbial communities [31–35], may provide a cheaper and more scalable method for the characterization of trunk-pathogen communities. This approach has already been applied to other pathosystems to address a variety of research objectives. For example, DNA metabarcoding has been used to identify candidate pathogens [36, 37] and potential biocontrol agents [38], to profile putative plant pathogens associated with insects [39], and to diagnose quarantine pathogens as part of national plant-protection programs [40–42]. DNA metabarcoding infers taxonomic composition of complex biological samples by amplifying, sequencing, and analyzing target genomic regions [43, 44]. The ribosomal ITS, which is under low evolutionary pressure and, thus, presents high levels of variation between closely related species, has been commonly used as a barcode for the analysis of fungal biodiversity [45, 46]. ITS is typically amplified by universal primers that anneal to the conserved flanking sequences. The “universality” of the primers, which derives from their ability to amplify a broad range of taxonomically unrelated species across the fungal Kingdom [47], is exploited in studies that aim to profile fungal communities, typically in exploratory analyses of environmental samples. We hypothesized that although universal primers may capture broad biodiversity in exploratory analyses, they may provide less accurate representation of microbial pathogen communities than primers that are designed and optimized to amplify species known to be associated with those communities, based on prior knowledge of disease etiology. After all, grapevine-trunk diseases are one of the most widely studied disease complexes in terms of species composition (Lamichane and Venturi, 2015). In this work, we designed and evaluated metabarcoding primers that were optimized to amplify the ITS regions of grapevine trunk pathogens. By a combination of in silico simulations, and analyses of ‘mock’ communities, samples from controlled inoculations, and samples from symptomatic vineyards, we demonstrated that community-customized metabarcoding provides both qualitatively and quantitatively a more accurate representation of trunk-pathogen communities than common universal primers.

## Results

### Primer design, selection, and validation with target species

**Fig. 1:**
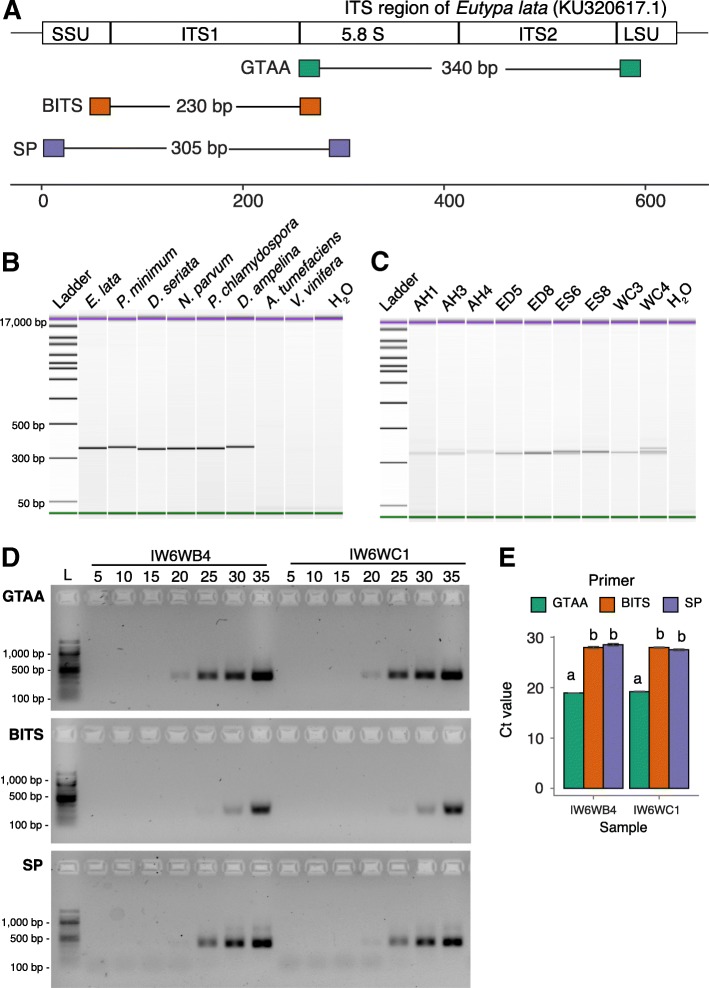
Primer design and testing. **a** Schematic representation of the annealing sites of forward and reverse GTAA, BITS, and SP primers in the fungal ribosal ITS. Reported amplicon sizes were calculated based on the ITS sequence of *Eutypa lata* (KU320617.1) as an example. **b** Bioanalyzer electropherograms showing PCR amplicons sizes generated using GTAA primers from purified fungal grapevine trunk pathogens: *E. lata, Phaeoa. minimum, Dip. seriata, N. parvum*, *Phaeom. chlamydospora,* and *Dia. ampelina*. *Agrobacterium tumefaciens*, *V. vinifera* and nuclease-free water were included as controls. **c** Bioanalyzer electropherograms showing PCR amplicons sizes generated using GTAA primers from selected field samples of mature vines infected with different trunk disease symptoms. AH: apparently healthy, ED: Eutypa Dieback, ES: Esca,WC: wood cankers (no leaf symptoms), and numbering corresponds to different biological replicates (same samples as in Fig. 5). **d** PCR products of GTAA, BITS, and SP primers from grapevine samples inoculated with *N. parvum* at six weeks post-inoculation sampled every five-cycles and visualized on an agarose gels. L: 100 bp Ladder. **e** Cycle thresholds (Ct) measured by qPCR of the same samples shown in (**d**)

We designed multiple degenerate primers that target the internal transcribed spacer (ITS) of grapevine trunk-associated ascomycetes (GTAA) using the TrunkDiseaseID as reference database [20]. Primer potential was determined in silico, considering the amplicon size and estimating the number of sequence hits to the database, their alignment mismatches, and gap scores. From a total of twenty forward and three reverse degenerate primers, primers GTAA182f and GTAA526r (GTAA, hereinafter) performed the best and were selected for further testing. While the BITS and SP primers target the ITS1 region, the selected GTAA primers target the entire ITS2 region with the forward and reverse primers aligning to the 5.8S ribosomal RNA and the large subunit ribosomal ribonucleic acid (LSU), respectively (Table 1 and Fig. 1a). The primers produced amplicons of approximately 350 bp from isolates of seven trunk pathogens, as expected based on the amplicon size predicted from the 213 ITS sequences of ascomycetes in the TrunkDiseaseID database (301.72 ± 7.53 bp; Fig. 1b). We obtained a similar amplicon size when the GTAA primers were used to amplify total DNA extracted from naturally infected grapevines with trunk-disease symptoms, including leaf symptoms of Esca or Eutypa dieback and wood cankers (Fig. 1c). Amplicon sequences matched the correct species, when aligned to our custom grapevine trunk pathogen-focused database using BLASTn, thereby confirming the ITS2 region amplified by the GTAA primers is informative for taxonomic assignments (Additional file 1). For field samples, multiple bands in Fig. 1c (e.g.: WC4) were likely caused by the size diversity among the ITS2 regions in the taxa amplified.

**Table 1:** Metabarcoding primer sequences targeting the ITS region used in this study

Primer name	Direction	Primer sequence 5′ to 3′	Citation
GTAA	Forward	AAAACTTTCAACAACGGATC	this study
GTAA	Reverse	TYCCTACCTGATCCGAGGTC	this study
BITS	Forward	CTACCTGCGGARGGATCA	[48]
BITS	Reverse	GAGATCCRTTGYTRAAAGTT	[48]
SP	Forward	CTTGGTCATTTAGAGGAAGTAA	[49]
SP	Reverse	GCTGCGTTCTTCATCGATGC	[49]

GTAA primer precision, sensitivity, efficiency, and usefulness for metabarcoding of grapevine trunk pathogens were compared to those of the BITS [48] and SP primers [49]. The BITS primers are widely used for fungal metabarcoding analysis in vineyards and grape must (e.g.: [50–54]), whereas the SP primers [49] were implemented in the Earth Microbiome Project (http://www.earthmicrobiome.org), and were used in recent microbial ecology studies [55–57] (Table 1 and Fig. 1a). Samples were from DNA extracted from potted grapevines either inoculated with *N. parvum* or from non-inoculated controls. By sampling PCR reactions every five cycles, the GTAA amplicon was visible on an agarose gel starting at 20 cycles, whereas those of SP and BITS were visible at 25 and 30 cycles, respectively (Fig. 1d). Furthermore, SP produced multiple bands, which may be due to non-specific binding and/or chimeric amplicons. Based on qPCR with the same samples, the average Ct values for GTAA were approximately nine cycles lower than those of BITS (*P* < 1.85e^− 04^) and SP (*P* < 3.50e^− 04^) (Fig. 1e). Overall, our findings suggest a higher affinity of the GTAA primers, when amplifying samples containing grapevine trunk pathogens.

### In silico simulation of amplification and taxonomic classification

**Fig. 2:**
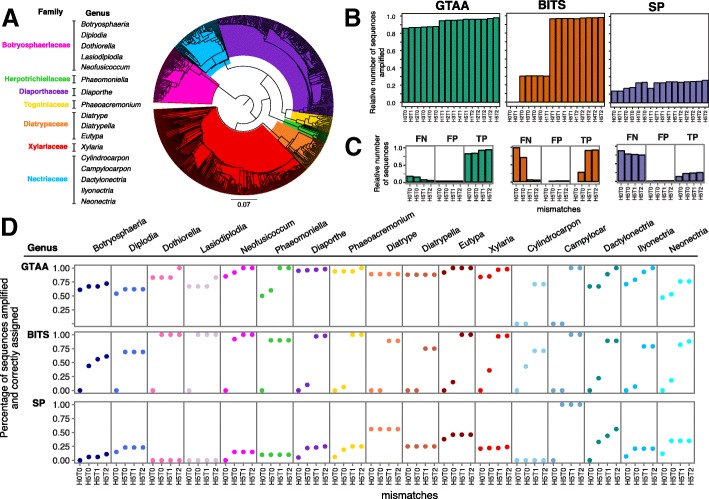
In silico simulation of amplification and taxonomic assignment. **a** Neighbor-joining tree of the full-length ITS sequences included in the custom database used in the simulation. **b** Barplots showing the number of sequences predicted to be amplified by each primer set at different combinations of mismatches. H: primer head. T: primer tail. Numbers correspond to the number of mismatches either in H or T. **c** Barplots showing the number of false negative (FN), false positive (FP), and true positive (TP) sequences with each primer set. **d** Percentage of sequences per genus correctly assigned with each primer set at the different mismatch combinations

We then carried out an in silico simulation that compared the potential amplification bias and taxonomic usefulness of GTAA, BITS, and SP primers. We compiled a custom database of 521 full-length ITS sequences across 17 genera of fungi commonly associated with grapevine trunk diseases (Fig. 2a, Additional file 2; [20]). We included only full-length ITS sequences to be able to compare primers that amplify different regions of the ITS (Fig. 1a). In silico amplification of each sequence in the custom database was carried out considering all alternative sequences of degenerated primers and allowing a series of mismatches between primer and template sequences. In silico amplification was carried out testing all possible combinations of allowed mismatches, from 0 to 5 mismatches in the first five nucleotides in 5′ of the primer and 0 to 2 mismatches in the remaining 3′ nucleotides of the primer. GTAA primers amplified a higher number of sequences than BITS and SP primers, for every parameter tested (Fig. 2b). When no mismatches between primer and target were allowed, GTAA primers amplified 85.80%, SP primers amplified 13.63%, and BITS primers were predicted to amplify none of the sequences in the database. When at least two mismatches were allowed in the tail of the primer, BITS and SP primers amplified only 16.70 and 30.33% of target sequences, respectively, whereas GTAA primers amplified 86.75%. With the most permissive parameters, GTAA primers amplified 98.08% of the sequences, and BITS and SP primers amplified 97.89 and 25.91%, respectively. The requirement of multiple mismatches for BITS primers to achieve a similar number of sequences as GTAA primers is consistent with the cycle-sampling results (Fig. 1d), and suggests that GTAA primers are more efficient than BITS at amplifying the ITS of grapevine trunk pathogens.

To determine if amplicons generated by GTAA primers are informative for taxonomic assignment, we analyzed with Mothur [58] the amplicons that were generated by the simulation using the 521 full-length ITS sequences as database. Mothur uses a *k*-mer based approach to compare the query to the database and assigns the query to the taxonomy with the highest probability. By comparing the assigned genera (observed) with the expected genera for each primer set we assessed false positive (FP; i.e., erroneously assigned), false negative (FN; i.e., not amplified or not assigned), and true positive (TP; i.e., correctly assigned, Fig. 2c) rate. We calculated rates of FP, FN, and TP for all combinations of mismatches to estimate the overall performance of each primer set. GTAA primers had the highest sensitivity (TP/(TP/FN)*100 = 89.50 ± 6.45%), followed by BITS (54.25 ± 47.86%), and SP (20.50 ± 2.53%). SP and GTAA primers displayed similar precision (SP: TP/(TP + FP)*100 = 97.50 ± 1.00%; GTAA: 97.00 ± 0.00%), which was higher than that of BITS primers (72.25 ± 48.18%). The different performance of the three primer sets in the simulation appeared to be mostly due to amplification bias against certain genera (Fig. 2d). GTAA primers amplified and correctly assigned to the proper genera a larger fraction of sequences than the other two primer sets for 14 out of 17 genera tested. This was the case for the following widely distributed trunk pathogens: *Eutypa* (GTAA: 98.0 ± 4.0%, BITS: 53.8 ± 53.8%, and SP: 44.0 ± 4.0%), *Diaporthe* (GTAA: 96.5 ± 1.3%, BITS: 51.3 ± 53.6%, and SP: 18.7 ± 9.3%), and *Phaeoacremonium* (GTAA: 95.5 ± 3.0%, BITS: 51.5 ± 56.1%, and SP: 18.7 ± 9.0%). BITS primers correctly assigned more sequences for *Lasiodiplodia* (GTAA: 71.0 ± 8.0%, BITS: 75.0 ± 50.0%, and SP: 0.0 ± 0.0%) and *Cylindrocarpon* (GTAA: 35.5 ± 41.0%, BITS: 46.3 ± 33.54%, and SP: 0.0 ± 0.0%). SP primers correctly assigned more sequences for *Campylocarpon* (GTAA: 50.0 ± 57.7%, BITS: 50.0 ± 57.7%, and SP: 75.0 ± 50.0%). Overall, this simulation predicted that, unlike the two universal primer sets, GTAA primers amplify ITS of more trunk pathogens and allow taxonomic assignment with greater sensitivity (i.e., higher true positive rate) and specificity (i.e., lower false negative rate). SP primers were not included in further experiments, due to their poor performance in these early stages.

### Analysis of mock communities and infection time course

**Fig. 3:**
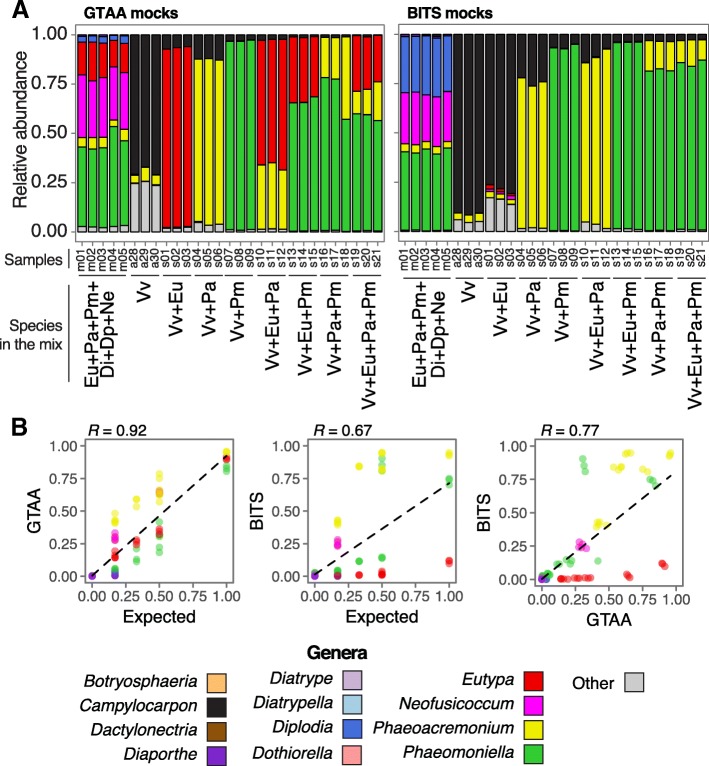
Results of DNA metabarcoding of mock communities. **a** Stacked barplots showing the relative abundance of genera in the mock communities with different proportion of fungal species identified using the GTAA and BITS primers. Eu: *Eutypa*, Pa: *Phaeoacremonium*, Pm: *Phaeomoniella*, Di: *Diaporthe*, Dp: *Diplodia*, Np: *Neofusicoccum,* and Vv: *Vitis vinifera.* **b** Linear correlations between observed and expected abundances for each genus contained in the mock community. R values correspond to *Pearson’s* correlation coefficients

To evaluate the primers for characterizing the species composition of mixed infections, we sequenced mock community samples with an Illumina MiSeq (Fig. 3a-b). Mock samples were created by mixing DNA of apparently healthy grapevine stems and fungal DNA as follows: 90% grape with 10% of fungal DNA *(E. lata*, *Phaeoa. minimum*, or *Phaeom. chlamydospora*), 80% grape with 10% of each of two fungal isolates, or 70% grape DNA with 10% of each of three fungal isolates. Another set of samples were created with equal concentrations of DNA of a total of six species (see more details in Methods and Additional file 3). We tested multiple approaches for taxonomic assignment. Although they all delivered very similar result, the OTU classifier Mothur was selected for further analysis because it overall delivered the greatest correlation between observed and expected values (Additional file 4). Although the DNA was extracted from stems with no symptoms of trunk disease, both primer sets detected fungi, mostly belonging to the genera *Campylocarpon* and *Phaeoacremonium* (Fig. 3a). In these samples, compared to the GTAA primers, the BITS primers assigned a greater proportion of reads to known taxa, likely because GTAA were designed to target taxa associated with trunk diseases. When grape DNA was mixed with DNAs of *Phaeoa. minimum* and *Phaeom*. *chlamydospora* both primer sets identified the correct taxa, with small relative difference from the expected values [relative difference between expected and observed (δ) in GTAA = 11.02 ± 7.0% and in BITS = 16.68 ± 11.39%]. For mock communities including *Eutypa*, GTAA primers detected this trunk pathogen in similar amounts to the expected abundance (δ = 9.74 ± 1.10%), whereas BITS primers underestimated its abundance (δ = 88.87 ± 1.27%). In mock communities with equal concentrations of DNA from *E. lata*, *Phaeoa. minimum*, *Phaeom*. *chlamydospora*, *N. parvum, D. seriata*, and *D. ampelina*, the correlation of expected and observed abundances in these mock communities was greater for GTAA (*R* = 0.92) than BITS (*R* = 0.67), though there was no significant difference between both primers (*P* = 0.16, Fig. 3b; Additional file 5). The BITS primers showed an underrepresentation of *Eutypa* (δ = 16.70 ± 0.12%), and *Phaeoacremonium* (δ = 13.10 ± 0.63%), and overrepresentation of *Phaeomoniella* (δ = 24.34 ± 1.56%) (Fig. 3a). Because DNA was mixed in equal amounts, the expected relative abundance of each genus was 16.6%. GTAA primers detected *Eutypa* at 16.42 ± 2.41%, whereas BITS primers detected this trunk pathogen at 0.05 ± 0.03%. In the case of *Diplodia*, GTAA primers estimated the abundance of the genus at 3.13 ± 0.48% and BITS primers at 28.9 ± 0.8%. Interestingly, neither primer set was able to detect properly *Diaporthe*, reporting only 0.58 ± 0.23% and 0.87 ± 0.15% for GTAA and BITS primers, respectively. Nonetheless, GTAA primers provide a better qualitative and quantitative representation of important trunk pathogens.

**Fig. 4:**
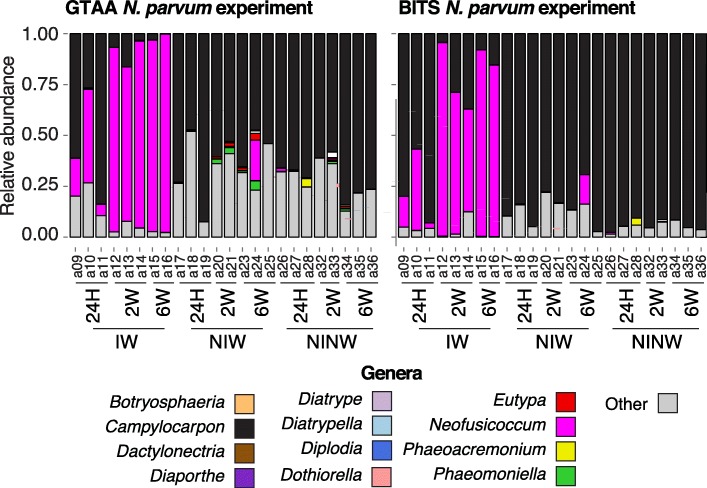
Results of DNA metabarcoding of an infection time-course. Stacked barplots showing the relative abundance of the genera detected in a time course experiment after inoculation with *N. parvum*. IW: wound-inoculated with *N. parvum*; NIW: non-inoculated non-wounded; NINW: non-inoculated non-wounded controls

We then tested the two primers using grape samples collected at different time points after controlled inoculation with a trunk pathogen. The objective of this analysis was to determine if the metabarcoding approach could detect quantitative differences between samples at early and late stages of infection. *Neofusicoccum parvum* was selected for this analysis as it is a fast growing and aggressive trunk pathogen and, therefore, can be used for studying grapevine stem colonization over a reasonable amount of time. Vines were inoculated with *N. parvum* and stem samples were collected at 24 h, 2 weeks, and 6 weeks post-inoculation. Plants non-inoculated wounded (NIW) and non-inoculated non-wounded (NINW) were included as controls. As expected, *Neofusicoccum* was predominant in the inoculated wounded (IW) samples, but absent from the controls (Fig. 4)*,* except for a single NIW sample, possibly due to cross-contamination during wounding or from contamination of the propagation material. The two primer sets provided similar fungal composition profiles characterized by five-fold increase in the average percentage of *Neofusicoccum* between 24 h and 6 weeks post-inoculation.

### Analysis of field samples and comparison with reference-based shotgun metagenome sequencing

**Fig. 5:**
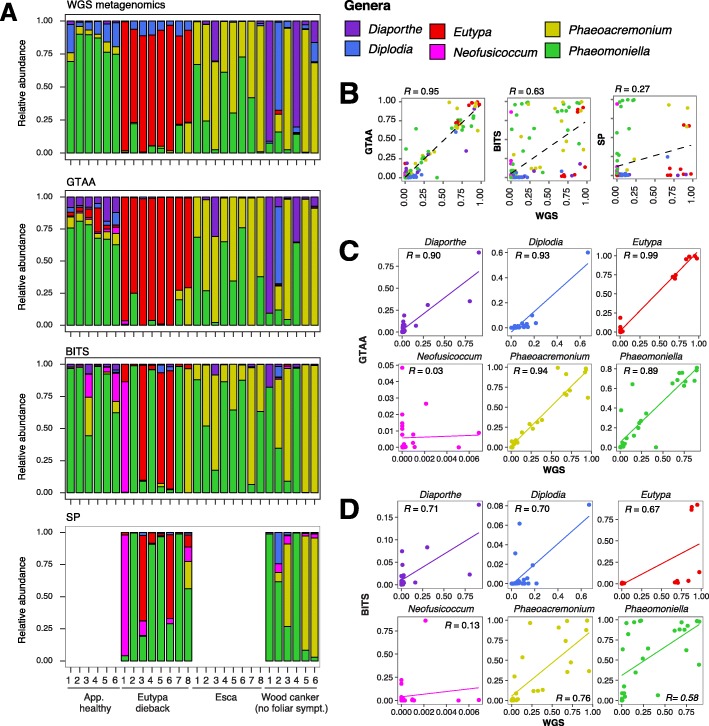
Results of DNA metabarcoding of field samples and comparisons with shotgun whole-genome metagenomics (WGS). **a** Stacked barplots showing the relative abundance of the genera detected by WGS and DNA metabarcoding with GTAA, BITS, and SP primers. The 28 samples were collected from mature vines in the field with the following trunk disease symptoms: apparently healthy (no foliar or wood symptoms), Eutypa Dieback, Esca and wood cankers (no leaf symptoms). The numbering corresponds to different biological replicates grouped by the disease symptoms displayed by the grapevines from where they were collected. **b** Scatter plots showing the correlations of the relative abundance obtained by DNA metabarcoding and WGS. **c** Scatter plots showing the correlations of the relative abundance separately for each genus obtained by DNA metabarcoding using the GTAA primers and WGS. **d** Scatter plots showing the correlations of the relative abundance separately for each genus obtained by DNA metabarcoding using the BITS primers and WGS. R values correspond to *Pearson’s* correlation coefficients

We then tested the primers on naturally infected grapevines. We used the same 28 field samples described in [13], which allowed us to compare the metabarcoding approach with the quantitative taxonomic profiles obtained by a reference-based shotgun metagenome sequencing. The samples were grouped according to symptoms into Eutypa dieback (ED), Esca (ES), wood canker without foliar symptoms (WC), and apparently-healthy (AH). All 28 field samples were amplified with both GTAA and BITS primers. A subset of 14 samples was amplified also using SP primers. Taxonomic classification was performed with multiple methods and results compared to the whole-genome metagenomics results, which, at least in case of GTAA, delivered very similar results (see more details in Methods and Additional file 6). Mothur results had the greatest correlation with shotgun metagenome sequencing for both primers and were used for further analyses. In addition to those with genomes in the multispecies reference, taxonomy assignment based on amplicon metabarcoding detected other 14 genera with abundances > 0.05% in one or more samples. Both GTAA and BITS primer sets identified *Alternaria*, *Cyphellophora*, and *Penicillium*, whereas *Cladosporium*, *Aureobasidium*, *Gibberella*, and *Cryptovalsa* were only identified by GTAA primers, and *Angustimassarina, Exophiala, Erysiphe, Meyerozyma, Acremonium*, and *Vishniacozyma* by BITS primers. GTAA primers revealed species abundances at very similar levels to those obtained by metagenomics analysis (Fig. 5a), with a strong linear correlation between the two approaches (*R* = 0.95; Fig. 5b), which was higher than those of both BITS (*R* = 0.63) and SP primers (*R =* 0.27). In agreement with the other results described above, BITS primers underestimated *Eutypa* in Eutypa-dieback samples and overrepresented *Phaeomoniella* in Esca samples. The even weaker correlation obtained with SP primers was due to the strong bias against *Eutypa* and *Diaporthe*. Surprisingly, *Diaporthe*, which was detected at much lower level than expected in the mock communities, showed a good correlation between metabarcoding and metagenomic results with both GTAA (*R =* 0.90) and BITS (*R =* 0.71) primers (Fig. 5c & d). This inconsistency confirms the difficulty in resolving this genus [59]. GTAA primers showed stronger correlations across all genera of trunk pathogens represented in the multi-genome reference (0.89 < *R <* 0.99, Fig. 5c) compared to those of BITS (0.58 < *R* < 0.75). Both primer sets overestimated *Neofusicoccum,* potentially because of the presence of other Botryosphaeriaceae, whose sequences may have been wrongly assigned to *Neofusicoccum* as ITS has been shown to poorly differentiate the species in this family [59]. Overall, our findings confirm the universal primers have a significant bias against important taxa and were outperformed by our GTAA primers for trunk pathogens.

## Discussion

In this study, we tested the application of DNA metabarcoding to profile the fungal taxa associated with grapevine trunk diseases. We show that DNA metabarcoding of ribosomal ITS amplified with commonly-adopted universal primers consistently misrepresented the abundance of important trunk pathogen species, such as *Eutypa* and *Phaeomoniella*. The customization of primer design using trunk pathogen sequences as template led to improve the results with greater sensitivity. This was likely due to greater sequence similarity between the GTAA primers and the ITS of the grapevine trunk pathogens they target. On average the sequence identity of the grapevine trunk pathogen targets was significantly greater with the GTAA primers (97.4 ± 5.5%; *P* < 2e-16) than with the other universal primers (BITS: 90.2 ± 7.1%; SP: 83.3 ± 0.2.2%) used in the study. The lower identity of the universal primers was likely responsible for the bias of the universal primers against important trunk pathogen taxa. Amplification bias of universal ITS primers due to higher levels of mismatches for certain taxonomic group has been observed in previous studies [35, 60–63]. Importantly, we also showed that the GTAA primers had higher sensitivity while maintaining a precision threshold for taxonomic assignment of 97%, suggesting that the choice of the target region within the ITS, in this case the ITS2, also played a role in improving the DNA metabarcoding for these organisms. We should stress out that BITS and SP primers are not the only available universal primers and the goal of this study was not to provide a comprehensive survey of all universal ITS metabarcoding primers. BITS and SP were selected, because they are both widely used DNA barcoding primers, including in studies conducted on vineyard and wine must samples [49–54]. We cannot rule out that other universal primers targeting the ITS1 or ITS2 regions that were not tested in this study may have performed differently. However, the results presented in this study show that universal primers may not be always appropriate to study a fungal community and, when fungal community composition is available, researchers should consider customizing their DNA metabarcoding primers. In addition, we illustrate the value of assessing both the amplification and taxonomy usefulness of the metabarcoding primers in silico prior to downstream wet lab evaluations.

In addition to customization of primers, the inclusion of other gene targets as additional DNA barcodes should help overcome some of the limitations associated with the ITS region, such as copy number variation between and within species and low resolution in separating some phylogenetically closely related fungal species [45, 64]. For example, the ITS region does not accurately identify species of plant-pathogenic fungi like *Alternaria*, *Botryosphaeria,* and *Diaporthe* [59]. The genera for which the GTAA primers consistently underestimated abundances like *Lasiodiplodia, Botryosphaeria, Diplodia,* and *Diaporthe* are known to be difficult to be resolved with the ITS region alone [59]. The high correlation between metagenomics and metabarcoding results using the GTAA primers suggest that copy number variation of the ITS region is not an overwhelming issue for the grapevine trunk pathogens present in the field samples.

Nonetheless, we expect that the inclusion of additional barcodes, such as β-tubulin and elongation factor 1-α, will help increase accuracy of taxonomic identification at the species level and help measure those genera for which the ITS is known not to be effective [20, 23, 65, 66].

## Conclusions

As trunk diseases are complex diseases caused by mixed infections, DNA metabarcoding should provide a rapid and effective method for high-throughput multispecies profiling, overcoming the limitations of currently applied methods. Universal primers are advantageous in exploratory analysis where a priori knowledge on the taxonomic composition of the samples is limited or not available. However, a more targeted approach should be used when the objective is to profile a more defined group of microorganisms, like the grapevine trunk pathogens which symptoms have been consistently associated with certain fungal species [4, 20, 67]. Overall, the results presented here demonstrated that DNA metabarcoding can be applied to grapevine trunk diseases. With further improvement of taxonomic identification by combining multiple barcoding loci and of quantification by measurement of direct correlation between fungal biomass and PCR amplification cycles, we envision DNA metabarcoding to be routinely applied in trunk pathogen research and profiling. DNA metabarcoding provides multiple advantages to methods employed in the past. Namely, there is no need of fungal isolation, it allows high number of samples to be analyzed at the same time given the multiplexing potential of the technology, and takes advantage of the constantly improving high-throughput sequencing technologies. Since wood pathogens may remain asymptomatic in young, non-stressed vines, propagation material may contain latent fungal infections and may become symptomatic after planting and serve as a source of inoculum for further infections of potentially clean plants. By allowing the rapid testing of large number of wood samples from mother plants in foundation blocks and propagation material in nurseries, we expect that the applications of metabarcoding to trunk pathogen profiling will help reduce the amount of trunk pathogens introduced into vineyards at planting as well as the incidence of young vine decline. Our results also demonstrated that primer customization and testing are crucial to ensure the validity of DNA metabarcoding results.

## Methods

### Metabarcoding primers targeting grapevine trunk-associated ascomycetes (GTAA)

Ribosomal Internal transcribed spacer (ITS) sequences of trunk pathogens and other wood-colonizing fungi of grape, specifically in the Division Ascomycota, were retrieved from the TrunkDiseaseID.org database [20]. Sequences were aligned using ClustalW2 (v2.1; [68]) to identify conserved regions. Sequence alignments of the conserved regions were used as input for the metabarcoding primer design software Primer Prospector v1.0.1 [69], using a sensitivity threshold of 80% and an initial primer seed size of 5 bp. The ITS sequence of *E. lata* (GeneBank KU721859.1) was used as a ‘reference’. The final primer set was selected based on median amplicon size, and mismatches, gaps, and numbers of matches to the sequences in the database. The base pairs ‘AG’ were used as a linker between the primer and an eight-nucleotide barcodes on the 5′ region of the forward primer sequence. Barcode sequences were as described in [70]. A list of barcoded forward GTAA primers is listed in Additional file 7.

A custom database was compiled with full length ITS sequences of species in the following genera commonly isolated from grapevines: *Botryosphaeria, Diplodia, Dothiorella, Lasiodiplodia, Neofusicoccum, Phaeomoniella, Diaporthe, Phaeoacremonium, Diatrype, Diatrypella, Eutypa, Xylaria, Cylindrocarpon, Campylocarpon, Dactylonectria, Ilyonectria*, and *Neonectria*. Sequences were retrieved from the NCBI GenBank repository. Completeness of the ITS sequences was validated using the hidden Markov models-based software ITSx [71]. Only sequences spanning the entire ITS region (ITS1, 5.8S, and ITS2) were kept for downstream analysis. Species and GenBank accessions of the complete ITS sequences included in the custom database are listed in Additional file 2. To reduce redundancy and identify outliers, the complete ITS sequences were clustered using the UCLUST algorithm [72] integrated in QIIME (v1.9.1; [73]) with 97% identity. The longest representative sequence of each cluster was selected, using the QIIME ‘pick_rep_set.py’ function. All representative sequences were aligned using Mafft v7.271 [74]) with the ‘--auto’ argument and 1000 iterations. Sequences clustering outside the expected family were removed from the final custom database.

The program Degenerate In-Silico PCR (dispr, https://github.com/douglasgscofield/dispr) was used to predict and evaluate the amplification of sequences of the custom ITS database, using our GTAA primers, and universal BITS [48], and SP [49] primers. Dispr allowed an amplicon size of 100 to 400 bp, all combinations up to five mismatches in the head of the primer (‘H’ or 5′-most region), and all combinations up to two mismatches in the tail of the primer (‘T’ or the remaining 3′-end of the primer). The resulting amplicons produced in silico were then used for taxonomy assignment with 80% confidence, using Mothur (v1.39.5; [58]), as it is integrated in QIIME (v1.9.1). The UNITE database v7.2 [75] was used as taxonomic reference. True positives were defined as sequences that were assigned to the expected genus, false positives were sequences assigned to a different genus, and false negatives were sequences not assigned to any genus or were not amplified by dispr.

To test primer affinity, quantitative real time PCR (qPCR) was performed using a QuantStudio 3 instrument (Applied Biosystems). Each 15 μl reaction mixture contained 2 ng of gDNA, 0.3 μM each primer and, 1X Power SYBR green PCR Master Mix (Applied Biosystems). The PCR stage included a first denaturation step (95 °C 10 min), followed by 40 cycles of 95 °C (15 s) and 60 °C (1 min).

To generate mock communities, we combined (i) DNA from a healthy grapevine with DNA from pure cultures of three trunk pathogens at different concentrations, or (ii) equal concentrations of DNA from pure cultures of six trunk pathogens. For the former, grape and fungal DNA were combined as follows: 90% grape with 10% *E. lata* isolate Napa209 [76], *Phaeoa. minimum* isolate 1119 [77], or *Phaeom. chlamydospora* isolate C42 [78]; 80% grape with 10% of each of two fungal isolates (in all three pair-wise combinations of the three isolates); and 70% grape DNA with 10% of each of the three fungal isolates. For the latter, equal concentrations of DNA were combined from the same three trunk pathogens and three additional species: *N. parvum* isolate UCD646So [11], *Dia. ampelina* isolate Wolf911 [79], and *Diplodia seriata* isolate SBen831 [80]. Grape DNA was extracted from the stems of a non-inoculated, non-wounded plant; this DNA template came from a previous experiment [81]. The mock communities containing grape DNA were amplified and sequenced as three independent samples, whereas the mock community of six fungal DNAs was amplified and sequenced as five independent samples. The fungi used to prepare the mock communities were grown on Potato Dextrose Agar (PDA; Difco laboratories, Detroit, MI). DNA was extracted as described in [73] and measured with a fluorometer (Qubit, Life technologies). To test *in planta* detection of a trunk pathogen at variable levels of infection (i.e., from low to high concentrations of fungal biomass over time), DNAs for the infection time course of *N. parvum* were extracted from the same samples described in [81]. Briefly, 1-year-old potted *V. vinifera* ‘Cabernet Sauvignon’ FPS 19 plants were inoculated with isolate UCD646So mycelia. Woody stems were collected at seven time points: 0 h post inoculation (hpi), 3 hpi, 24 hpi, 2 weeks post inoculation (wpi), 6 wpi, 8 wpi, and 12 wpi. Wood samples from 1 cm below the inoculation site were collected using flame-sterilized forceps and immediately placed in liquid nitrogen for nucleic acids extraction. Infections were confirmed by positive recovery of the pathogen after 5-day growth on PDA.

To test *in planta* detection of multiple trunk pathogens in mixed infection (i.e., to characterize the species composition of a naturally established trunk-pathogen community), DNA from the same 28 field samples described in [13] was used to make cross-technology comparisons. These field samples were collected from mature vines (> 8 years-old) showing a variety of the most common symptoms associated with trunk diseases. Wood samples were collected from distinct plants with the following combinations of symptoms: Eutypa dieback foliar and wood symptoms, Esca foliar and wood symptoms, wood symptoms and no foliar symptoms, and apparently healthy plants with no foliar or wood symptoms.

### High throughput sequencing libraries

Each sample was amplified using the unique 8-nt barcode forward primer sets for GTAA and BITS, to enable sample multiplexing. The 25-μl PCR reaction mix contained 2 ng of DNA template, 1X Colorless GoTaq flexi buffer (Promega Corporation, Madison, WI), 1.5 mM MgCl_2_, 0.1 mg/ml BSA, 0.2 mM dNTPs, 0.4 μM of each primer, and 1.25 units of GoTaq Flexi DNA polymerase (Promega Corporation, Madison, WI). PCR program (Veriti thermal cycler, Applied Biosystems) was as follows: initial denaturation at 94 °C for 3 min, followed by 35 cycles at 94 °C for 45 s, 55 °C for 1 min., and 72 °C for 1 min., and a final extension at 72 °C for 10 min. In the experiments to assess primer affinity, reactions were stopped after 5, 10, 15, 20, 25, 30, and 35 cycles. Following PCR, amplicon size and uniqueness were verified using gel electrophoresis, and bands were cleaned using Ampure XP magnetic beads (Agencourt, Beckman Coulter). DNA concentration was determined for each purified amplicon using Qubit (Life technologies). For the single isolate validation, amplicons were sequenced with Sanger (DNA Sequencing Facility, University of California, Davis).

For high-throughput sequencing, equimolar amounts of all barcoded amplicons were pooled into a single sample, the total concentration of which was determined by Qubit. Five hundred nanograms of pooled DNA were then end-repaired, A-tailed and single-index adapter ligated (Kapa LTP library prep kit, Kapa Biosystems). After adapter ligation, the sample was size-selected with two consecutive 1X bead-based cleanups; concentration and size distribution were determined with Qubit and Bioanalyzer (Agilent Technologies), respectively. DNA libraries were submitted for sequencing in 250-bp paired-end mode on an Illumina MiSeq (UCDavis Genome Center DNA technologies Core). All FASTQ files with the amplicon sequences separated by barcode were deposited in the NCBI Sequence Read Archive (BioProject: PRJNA485180; SRA accession: SRP156804).

### Amplicon sequencing community analysis

Adapter-trimming was carried out using BBDuk (BBMap v.35.82; http://jgi.doe.gov/data-and-tools/bb-tools/) in paired-end mode with sequence “AGATCGGAAG” and the following parameters: ktrim = r, k = 10, mink = 6, edist = 2, ordered = t, qtrim = f and minlen = 150. Adapter-trimmed FASTQ files were then quality-filtered using Trimmomatic v0.36 [82] with paired-end mode, phred33, a sliding windows of 4:19, and a minimum length of 150 bp. Sequencing data were then processed in the QIIME environment v1.9.1 [73]. Barcodes were extracted from the FASTQ files using the “extract_barcodes.py” function with the “-a” argument that attempts read orientation and a barcode length of eight base pairs. The resulting sequences and barcodes were used to tag the reads with “split_libraries_fastq.py”, a threshold quality score of 20, and a barcode size of eight basepairs. Operational taxonomic units (OTUs) were identified with a 99% similarity threshold using the UCLUST algorithm [72] with the reverse strand match enabled (“-z”), and the longest sequence of each OTU was chosen as representative sequence. Taxonomy assignment was carried out using Mothur (v1.39.5; [58] with the UNITE database v7.2 [75] or the custom database as reference with a 80% confidence threshold). Two additional OTU classifiers, BLAST and UCLUST, as implemented in QIIME were also used to analyze mock samples and field samples for taxonomy assignment testing inside QIIME (Additional files 4 and 6). For each sample, sequences were randomly sampled with the function “single_rarefaction.py” from the OTU tables to obtained a total number of sequences per sample equal to the lowest number of reads across GTAA, BITS, and SP datasets. In the mock and *N. parvum* artificially inoculated samples, the lowest value corresponded to 1119 reads of the sample “a33” from the GTAA primers. In the field samples the value corresponded to 23,609 reads of sample “ED6” from the BITS primers. The Taxonomy tables at the genus level were then created using “summarize_taxa.py”. Taxonomic assignments based on OTUs generated in QIIME were compared with results obtained by BLASTn (v2.2.31+) alignments of each individual read to the custom ITS database. We considered as hits only those alignments that covered more than 97% of the query sequence with sequence identity greater than 97%. The sequence with the highest identity was chosen as the best hit for each query sequence. The results were compared to the expected values and presented in Additional file 4.

## Additional files

Additional file 1: **Text S1.** Assembled amplicon sequences produced by the GTAA primers. Amplicons were sequenced with Sanger. Each sequence description includes the GenBank accession number to the best hit of the custom database created in this study as well as identity and coverage of the alignment. (FASTA 2 kb)

Additional file 2: **Table S1.** Table with GenBank accession numbers and sequences retrieved from the NCBI used as a database for primer evaluation. TP: True Positive, FP: False Positive and FN: False Negative. (XLSX 124 kb)

Additional file 3: **Table S2.** Table with the proportions of fungal DNA of pure cultures of six grapevine trunk pathogens mixed to create the mock samples. Samples m01 to m05 were created with a mix of equal proportions of the six species. Samples s01 to s21 were created with a mix of 10% of one or more species, and a remaining percentage of grape DNA. The values presented are the expected proportions of fungal DNA. (XLSX 9 kb)

Additional file 4: **Figure S1.** Scatterplots showing the correlation between expected relative abundance based on how mock communities were prepared and the observed relative abundance of fungal taxa detected in the mock communities using the GTAA and BITS primers. The genus abundances resulting from the QIIME pipeline with Mothur, BLASTn and UCLUST taxonomy classifiers, as well as direct BLASTn of the reads to the custom database created in this study, were compared to the expected values. R values correspond to *Pearson’s* correlation coefficients. (PDF 643 kb)

Additional file 5: **Figure S2.** Scatterplots showing the correlation between expected relative abundances and observed values using GTAA and BITS primers for individual fungal taxa in the mock communities. R values correspond to *Pearson’s* correlation coefficients. (PDF 286 kb)

Additional file 6: **Figure S3.** Scatterplots showing the correlation of relative abundance of fungal taxa detected using GTAA and BITS, and whole-genome shotgun metagenomics results with multiple taxonomy classifiers. The genus abundances resulting from the QIIME pipeline with Mothur, BLASTn, and UCLUST, were compared to the metagenomics values. Mothur results were also compared to the other classifying methods. R values correspond to *Pearson’s* correlation coefficients. (PDF 634 kb)

Additional file 7: **Table S3.** List of forward GTAA primers with linker and barcode sequences. (XLSX 46 kb)
