# Supplementary material for: Profiling grapevine trunk pathogens in planta: a case for community-targeted DNA metabarcoding
Source: BMC Microbiol. 2018 Dec 14;18:214. doi: 10.1186/s12866-018-1343-0 (PMC6295080; doi:10.1186/s12866-018-1343-0)
Supplement: Supplementary file 4 — Figure S1. Scatterplots showing the correlation between expected relative abundance based on how mock communities were prepared and the observed relative abundance of fungal taxa detected in the mock communities using the GTAA and BITS primers. The genus abundances resulting from the QIIME pipeline with Mothur, BLASTn and UCLUST taxonomy classifiers, as well as direct BLASTn of the reads to the custom database created in this study, were compared to the expected values. R values correspond to Pearson’s correlation coefficients. (PDF 643 kb) [file 12866_2018_1343_MOESM4_ESM.pdf]

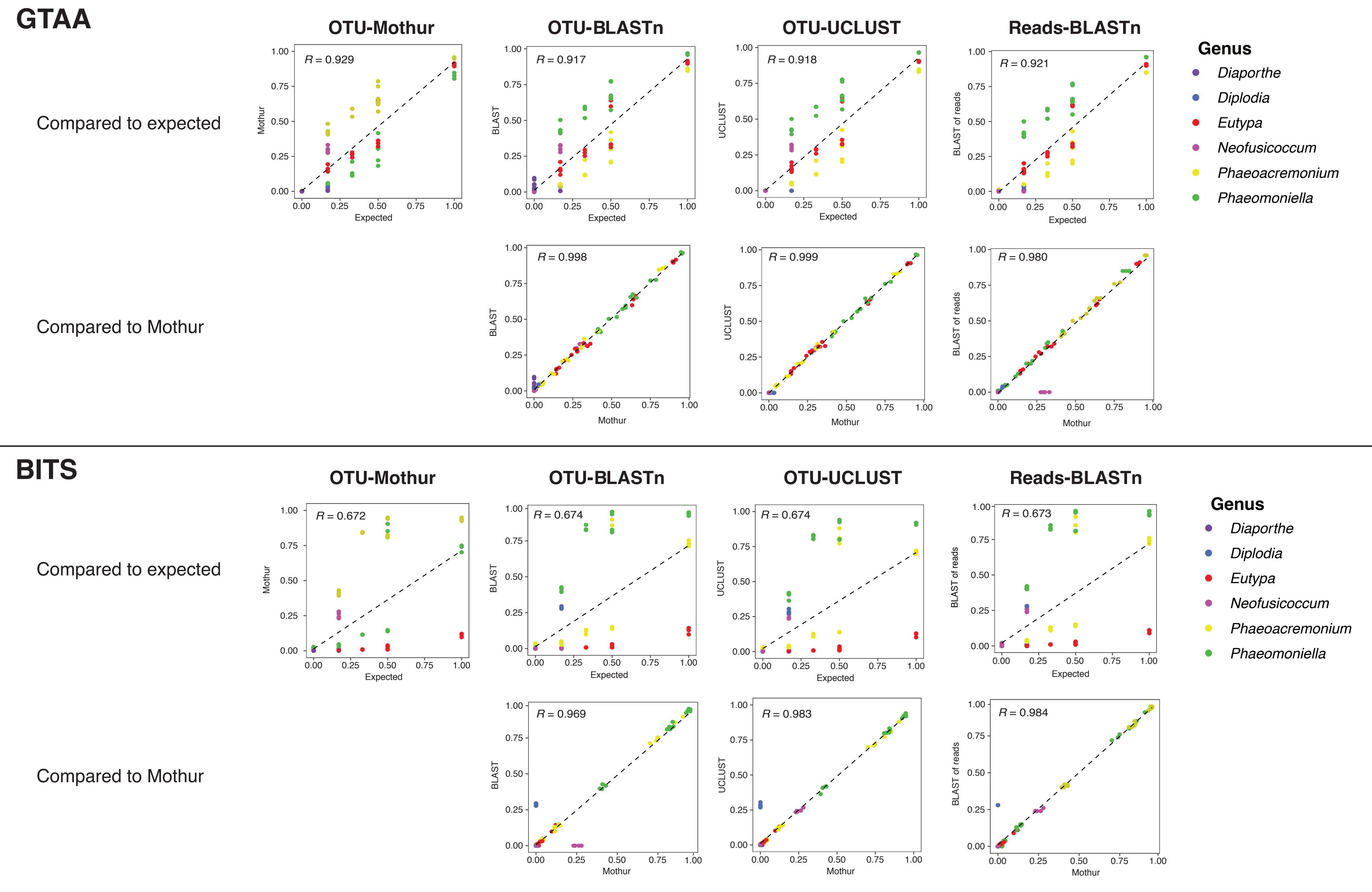

GTAA
OTU-Mothur
OTU-BLASTn
OTU-UCLUST
Reads-BLASTn
Compared to expected
R = 0.929
R = 0.917
R = 0.918
R = 0.921
Compared to Mothur
R = 0.998
R = 0.999
R = 0.980
BITS
OTU-Mothur
OTU-BLASTn
OTU-UCLUST
Reads-BLASTn
Compared to expected
R = 0.672
R = 0.674
R = 0.674
R = 0.673
Compared to Mothur
R = 0.969
R = 0.983
R = 0.984
Mothur
BLAST
UCLUST
BLAST of reads
Expected
0.00
0.25
0.50
0.75
1.00
Genus
Diaporthe
Diplodia
Eutypa
Neofusicoccum
Phaeoacremonium
Phaeomoniella
