# Supplementary figures and images for: Profiling grapevine trunk pathogens in planta: a case for community-targeted DNA metabarcoding
Source: BMC Microbiol. 2018 Dec 14;18:214. doi: 10.1186/s12866-018-1343-0 (PMC6295080; doi:10.1186/s12866-018-1343-0)

GTAA

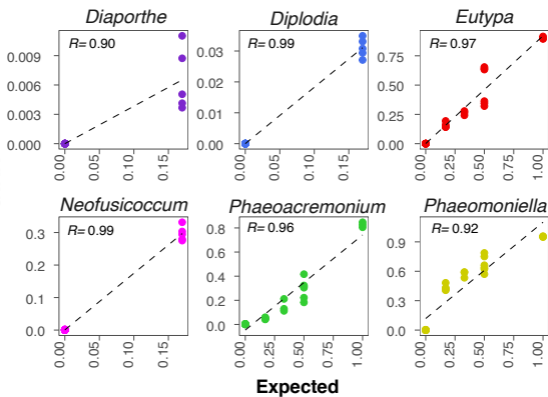

BITS

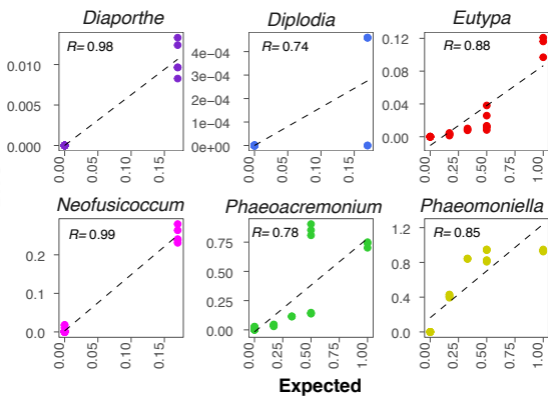

BITS

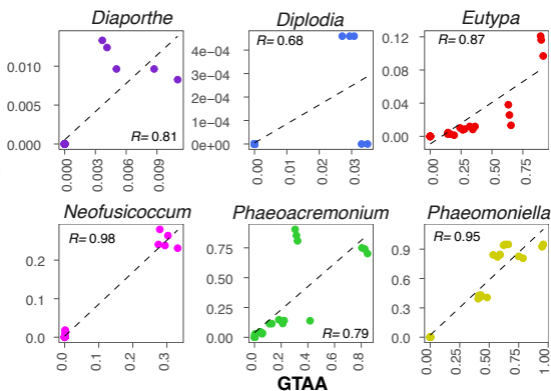

Supplement: Supplementary file 5 — Figure S2. Scatterplots showing the correlation between expected relative abundances and observed values using GTAA and BITS primers for individual fungal taxa in the mock communities. R values correspond to Pearson’s correlation coefficients. (PDF 286 kb) [file 12866_2018_1343_MOESM5_ESM.pdf]
