# Supplementary material for: Profiling grapevine trunk pathogens in planta: a case for community-targeted DNA metabarcoding
Source: BMC Microbiol. 2018 Dec 14;18:214. doi: 10.1186/s12866-018-1343-0 (PMC6295080; doi:10.1186/s12866-018-1343-0)
Supplement: Supplementary file 6 — Figure S3. Scatterplots showing the correlation of relative abundance of fungal taxa detected using GTAA and BITS, and whole-genome shotgun metagenomics results with multiple taxonomy classifiers. The genus abundances resulting from the QIIME pipeline with Mothur, BLASTn, and UCLUST, were compared to the metagenomics values. Mothur results were also compared to the other classifying methods. R values correspond to Pearson’s correlation coefficients. (PDF 634 kb) [file 12866_2018_1343_MOESM6_ESM.pdf]

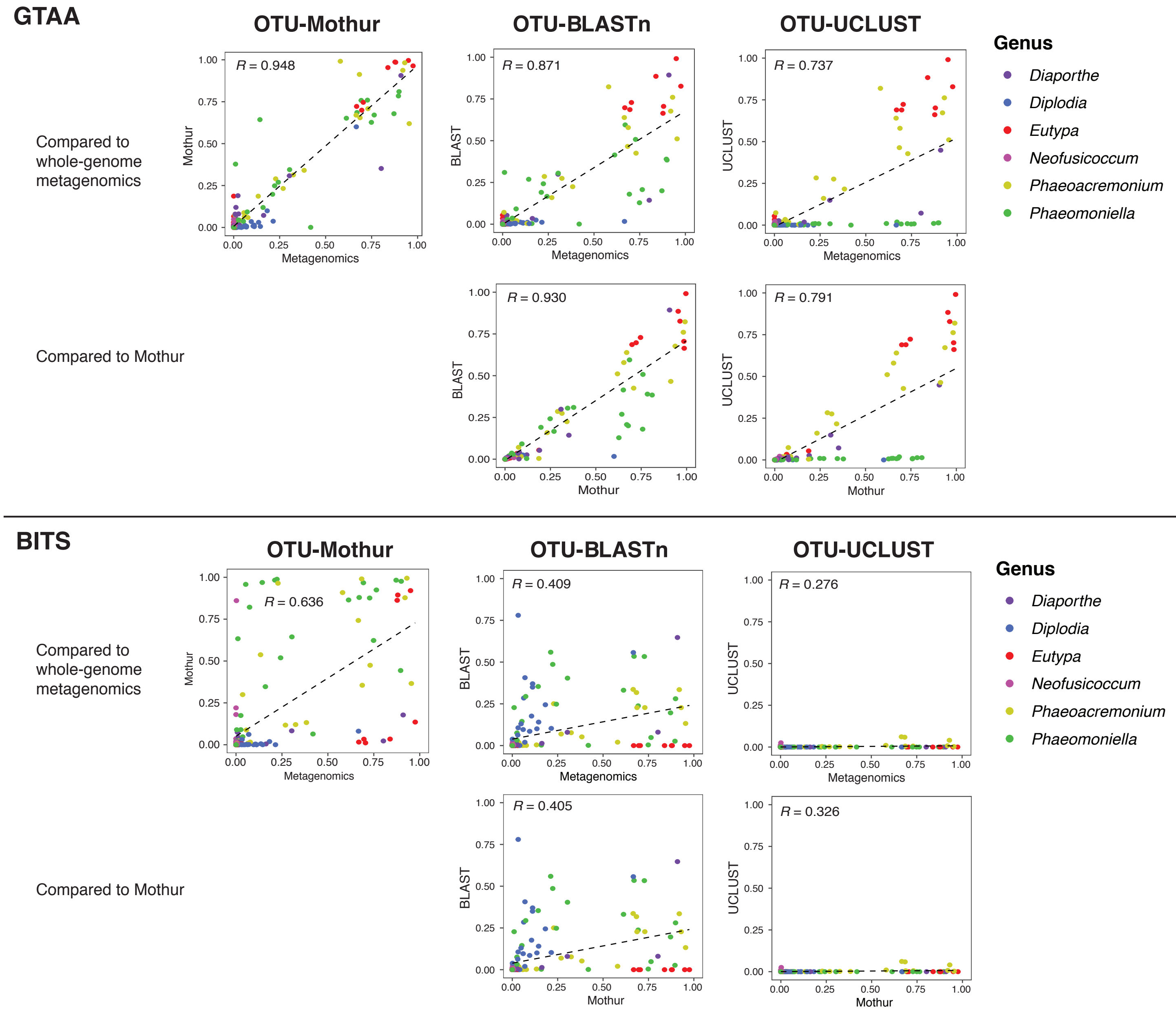
GTAA
OTU-Mothur
OTU-BLASTn
OTU-UCLUST
Genus
Diaporthe
Diplodia
Eutypa
Neofusicoccum
Phaeoacremonium
Phaeomoniella
Compared to whole-genome metagenomics
Compared to Mothur
R = 0.948
R = 0.871
R = 0.737
R = 0.930
R = 0.791
Mothur
BLAST
UCLUST
Metagenomics
BITS
R = 0.636
R = 0.409
R = 0.276
R = 0.405
R = 0.326
